# Genetic Diversity of *Rice stripe necrosis virus* and New Insights into Evolution of the Genus *Benyvirus*

**DOI:** 10.3390/v13050737

**Published:** 2021-04-23

**Authors:** Issiaka Bagayoko, Marcos Giovanni Celli, Gustavo Romay, Nils Poulicard, Agnès Pinel-Galzi, Charlotte Julian, Denis Filloux, Philippe Roumagnac, Drissa Sérémé, Claude Bragard, Eugénie Hébrard

**Affiliations:** 1 Earth and Life Institute, Applied Microbiology-Phytopathology, Université Catholique de Louvain (UCLouvain), Croix du Sud 2 Bte L07.05.03, 1348 Louvain-la-Neuve, Belgium; issiaka.bagayoko@uclouvain.be (I.B.); gromay@gmail.com (G.R.); claude.bragard@uclouvain.be (C.B.); 2 Consejo Nacional de Investigaciones Científicas y Técnicas (CONICET), Buenos Aires 1425, Argentina; marcoscelli@hotmail.com; 3 Instituto de Patología Vegetal (IPAVE, CIAP, INTA), Camino 60 cuadras Km 5, Cordoba 5119, Argentina; 4 PHIM, Plant Health Institute, Université de Montpellier, IRD, INRAE, CIRAD, SupAgro, 911 Avenue Agropolis, 34394 Montpellier, France; nils.poulicard@ird.fr (N.P.); agnes.pinel@ird.fr (A.P.-G.); charlotte.julian@cirad.fr (C.J.); denis.filloux@cirad.fr (D.F.); philippe.roumagnac@cirad.fr (P.R.); 5 CIRAD, UMR PHIM, Campus International de Montferrier-Baillarguet, 34398 Montpellier, France; 6 Laboratoire de Laboratoire de Virologie et de Biotechnologies Végétales, INERA—Institut de l’Environnement et de Recherches Agricoles, LMI Patho-Bios, Ouagadougou 01 BP 476, Burkina Faso; drissa.sereme@coraf.org

**Keywords:** RSNV, benyvirus, diversity, recombination

## Abstract

The rice stripe necrosis virus (RSNV) has been reported to infect rice in several countries in Africa and South America, but limited genomic data are currently publicly available. Here, eleven RSNV genomes were entirely sequenced, including the first corpus of RSNV genomes of African isolates. The genetic variability was differently distributed along the two genomic segments. The segment RNA1, within which clusters of polymorphisms were identified, showed a higher nucleotidic variability than did the beet necrotic yellow vein virus (BNYVV) RNA1 segment. The diversity patterns of both viruses were similar in the RNA2 segment, except for an in-frame insertion of 243 nucleotides located in the RSNV tgbp1 gene. Recombination events were detected into RNA1 and RNA2 segments, in particular in the two most divergent RSNV isolates from Colombia and Sierra Leone. In contrast to BNYVV, the RSNV molecular diversity had a geographical structure with two main RSNV lineages distributed in America and in Africa. Our data on the genetic diversity of RSNV revealed unexpected differences with BNYVV suggesting a complex evolutionary history of the genus *Benyvirus*.

## 1. Introduction

Rice is one of the major staple foods for global consumption and plays a crucial role in food security in developing countries [1]. More than 15 viruses have been reported to exist around the world, causing high yield losses. While most of these viruses are distributed in Asia, only two have recurrently been reported in Africa and America, i.e., the rice yellow mottle virus (RYMV) in Africa and the rice hoja blanca virus (RHBV) in America. However, other rice viruses have spread to those two continents. For instance, the rice stripe necrosis virus (RSNV) has recently emerged in America and is re-emerging in Africa. Hence, while RSNV was initially identified in the Ivory Coast in 1983 [2] and almost undetected since then, characteristic symptoms of crinkling yellow and deformation of rice leaves were recently observed in West-African countries, including Burkina Faso [3], Benin [4], Mali [5] and Sierra Leone [6]. The incidence of disease ranges locally from 37% to 80% in these African countries. In South and Central America, the virus has been episodically reported in several countries, including Colombia, Brazil, Ecuador, Panama and Argentina [7,8,9,10,11], causing severe epidemics leading to up to 40% yield losses [10].

Rice stripe necrosis virus belongs to the genus *Benyvirus*, family *Benyviridae* [12]. The type member of the genus, beet necrotic yellow vein virus (BNYVV), causes rhizomania, one of the most important diseases affecting sugar beet [13]. Distributed worldwide, BNYVV is responsible for yield losses of up to 50%. In addition to RSNV and BNYVV, two other benyviruses have been characterized so far, beet soil-borne mosaic virus (BSBMV), present in the USA in sugar beet, but without inducing symptoms on roots [14,15] and burdock mottle virus (BdMV) described in Japan in the case of burdock plants (*Arctium lappa*) [16].

Benyviruses have non-enveloped multipartite rod-shaped particles. They are transmitted by soil-borne plasmodiophorid protists of the genus *Polymyxa*. Benyviruses can persist in the soil for decades through the vector spores [17]. Benyviruses have positive single-stranded RNA genomes composed of two to five segments, depending on the viral species [12]. The housekeeping genes are encoded by the two largest segments of ca. 6600 and 4600 nucleotides, respectively. RNA1 contains a single large open reading frame (ORF) that encodes the replication polyprotein including methyltransferase (Mtr), helicase (Hel), protease (Pro) and the RNA dependent RNA polymerase (RdRp) domains [12]. RNA2 contains six ORFs (ORF2-ORF7). ORF2 encodes the major coat protein (CP). ORF3 is translated from a readthrough (RT) mechanism producing a fusion of the CP and RT domain embedded in the viral capsid and participates in the vector transmission [12]. ORF4-6 form the Triple Gene Block (TGB) involved in virus movement. TGBp1 binds to RNA and interacts with TGBp2 and TGBp3 [18]. ORF7 encodes the cysteine-rich protein (CRP) dedicated to the viral suppressor of RNA silencing (VSR) [19]. BNYVV and BSBMV clones have been constructed using RNA1 and RNA2 segments and have the ability to infect *Nicotiana benthamiana* and *Beta macrocarpa* [20,21]. However, in nature, BNYVV, BSBMV and BdMV have additional small genomic components (RNA3 and RNA4). RNA3 contains two ORFs that encode a p25 protein responsible for the modulation of symptoms in leaves and roots, and a 6.8 kDa protein expressed only from RNA3 truncated forms [22,23]. RNA4 encodes a p31 protein, described as a root specific VSR [20], and is involved in vector transmission [20,24]. RNA5 is only present within some BNYVV isolates, and encodes a p26 protein, which induces severe rhizomania symptoms and highly-reduced sugar yields [25]. It has been suggested that RNA5 originates from a duplication of RNA3 [26].

More than 110 isolates of BNYVV have been partly sequenced, and several genetic groups have been described [27,28,29]. Molecular analyses have been mainly focused on small segments 3, 4 and 5 and on the *cp* gene (RNA2). Up to now, only nine entire sequences of BNYVV RNA1 and RNA2 are publicly available. This lack of knowledge originated from the major role of the small segments in rhizomania symptoms, but also on the low diversity observed from the nine RNA1 and RNA2 sequences [30]. However, the housekeeping genes contained by both RNA1 and RNA2 segments are crucial, and even sufficient for RSNV, to fulfil the viral cycle of benyviruses. Until 2019, only a single whole RSNV genome (RNA1+RNA2) of a Colombian isolate was made publicly available in GenBank. Recently, four additional genomes from Brazil have been released, likely linked to the emergence of RSNV in South America, and to the advances in high-throughput sequencing (HTS) technologies. In addition, our team released two sequences from Mali. The aim of this study was to fill the knowledge gap on the genetic diversity of RSNV using a combination of classical Sanger and Illumina sequencing. As a result, we obtained nine new entire genomes. The sequences from Burkina Faso, Sierra Leone and Benin constitute with those from Mali, the first corpus of African genomic sequences. This large amount of data has allowed new genetic diversity analyses and comparisons at intra- and inter-specific levels, revealing unexpected features of *Benyvirus*.

## 2. Materials and Methods

### 2.1. Viral Isolates

Seven samples were collected between 2016 and 2018 in Argentina (SF2.1 and Cor18), Burkina Faso (BF01Bama, BF02Bama and BF03Bama) and Mali (ML01San and ML02San) (Table 1). The isolates from Argentina originated from irrigated rice plots in Corrientes Province and from pluvial rice fields in the Santa Fe Province. The isolates from Mali originated from lowland rice fields in the San region, and from pluvial rice plots at Bama in Burkina Faso. Four rice samples previously collected between 2014 and 2019 from Benin (Be2) [4], Mali (M1-1 and M2-1) [5] and Sierra Leone (SL254) [6] were also used in this study. Specifically, Malian isolates have been maintained under greenhouse conditions in separate tube cultures of rice var. Kogoni 91-1 infected with its vector *P. graminis*.

### 2.2. Genomic Sequencing

Different strategies were used to obtain the genomic sequences of the RSNV isolates. Total RNA extractions were performed from leaves or roots using the RNeasyPlant Mini kit (Qiagen) or TRIzol reagent (Invitrogen), according to manufacturers’ instructions. The sequence from Benin was amplified using primers at the 5’ and 3’ ends of the two RNA segments (Table S1) followed by library constructs according to the protocol recommended in [31]. Sequencing was performed by Illumina, and assembly was performed using Galaxy workflows [32,33], implemented on the South Green bioinformatic platform as described in [34]. The sequence Cor18 from Argentina was obtained using VANA-NGS (Virion-Associated Nucleic Acids—Next-Generation Sequencing, [35,36]) sequenced by Illumina, and assembled in Geneious software v.9.1.8 (Biomatters Limited, Auckland, NewZealand). The genomes of the other nine isolates were amplified by RT-PCR using different combinations of specific primers (Table S1), followed by sequencing by the Sanger method, and assembled using BioEdit software [37]. For the isolates from Burkina Faso and Mali, PCR products from the 5′ and 3′ ends of the genome were sequenced after cloning into pGEM-T Easy Vector (Promega, Madison, WI, USA), while internal amplicons were directly sequenced by the Sanger method.

### 2.3. Sequence Analyses

The eleven RNSV sequences obtained in this study, the reference genome from Colombia, and the four newly-released sequences from Brazil and available in the GenBank database [38] (Table 1) were aligned using MUSCLE in SEAVIEW v4.7 software [39]. Conserved functional domains of benyviruses were located along the sequences [40]. Nine BNYVV entire publicly available sequences were used for diversity comparison with RSNV (accession numbers RNA1 and RNA2, respectively: HM126464 and HM11790, MH106726 and MH106727, KX665536 and KX665537, KM434313 and KM434314, EU330453 and EU330452, EU330450 and EU330451, X05147 and X04197, D84410 and D84411, MT227164 and MT227165). The location of hydrophobic clusters and predicted disordered domains in TGBp1 were analyzed using HCA v1.0.2 (https://mobyle.rpbs.univ-paris-diderot.fr/cgi-bin/portal.py accessed on 18 September 2020) [41] and PONDR^®^ VL-XT (http://pondr.com accessed on 18 September 2020) [42,43], respectively. The presence of nucleolar localization signals (NoLS) and nuclear localization signals (NLS) was searched for in TGBp1 using a nucleolar localization sequence detector (NOD) [44] and a cNLS mapper, available online for prediction of NLS specific to the importin αβ pathway [45]. Helicase conserved motifs I-VI were located in the TGBp1 of RSNV, BNYVV, BSBMV, BdMV, potato mop-top virus (PMTV), peanut clump virus (PCV) and barley stripe mosaic virus (BSMV) (accession numbers: EU099845, MK170454, MT227165, JF513083, AB818899, MK539869, L07269 KY615805) based on [46]. Diversity analyses were performed independently for RSNV and BNYVV using DNAsp software, version 6.12.03 with default parameters [47] (Table S2). The location of parsimony informative polymorphisms was observed along the genome. Putative recombination events were identified using seven methods (RDP, GeneConv, Chimaera, MaxChi, BootScan, SiScan, and 3Seq) implemented in the RDP4 software, version 4.974 [48]. Only recombination events detected by at least three methods were considered and sequence alignments with recombinant regions removed were generated for phylogenetic analyses. Maximum-likelihood (ML) phylogenetic trees were reconstructed using the PHYML-3.1 algorithm implemented in SEAVIEW v4.7 [39] under the best-fitted nucleotide substitution model (TN93+G, K2+G, K2+I for the complete genome, the helicase and the CP datasets, respectively), with gap treatment by pairwise deletion and with 100 bootstrap replications. Phylogenetic trees were drawn using FigTree v1.3.1 (http://tree.bio.ed.ac.uk/software/figtree/ accessed on 17 September 2020).

## 3. Results

To date, five RSNV genomes have been entirely sequenced: one from the reference isolate collected in 1999 in Colombia [7], and four from isolates collected in 2019–2020 in Brazil. In this study, eleven new RSNV isolates were fully sequenced including the sequences from Mali recently released (Table 1). Two isolates from Argentina were collected in the major rice producing areas in Corrientes and Santa Fe Provinces. Nine isolates were originated from Mali, Burkina Faso, Benin and Sierra Leone. This dataset constitutes the first corpus of African RNSV genomic sequences.

**Figure 1:**
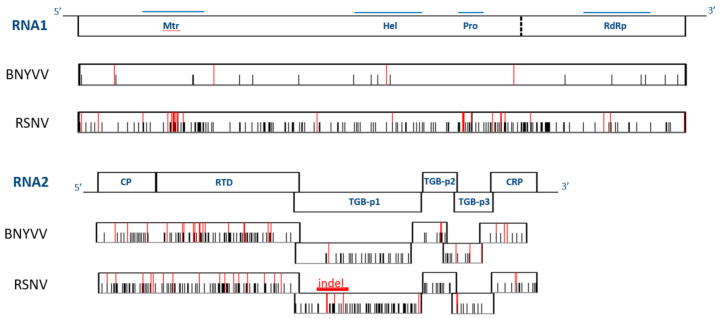
Polymorphisms along the RNA1 and RNA2 segments of RSNV and BNYVV. Black horizontal lines indicate untranslated regions, rectangular boxes indicate ORFs and blue horizontal lines indicate conserved functional domains. Black and red vertical bars indicate positions of parsimony informative polymorphisms (synonymous and non-synonymous polymorphisms, respectively), based on DNAsp analyses independently for each viral species [47]. Red horizontal line indicates the insertion in RNSV TGBp1. Mtr, methyltransferase, Hel, helicase, Pro, protease, RdRp, RNA-dependent RNA polymerase, CP, coat protein, RTD, read-through domain, TGBp, triple gene block protein, CRP, cystein-rich protein.

All RSNV full-length sequences showed the genomic organization of benyviruses (Figure 1). RNA1 segment encodes the replication polyprotein including Mtr, Hel, Pro and RdRp. RNA2 segment harbors six open reading frames encoding: CP, RTD, TGBp1, TGBp2, TGBp3 and CRP.

**Figure 2:**
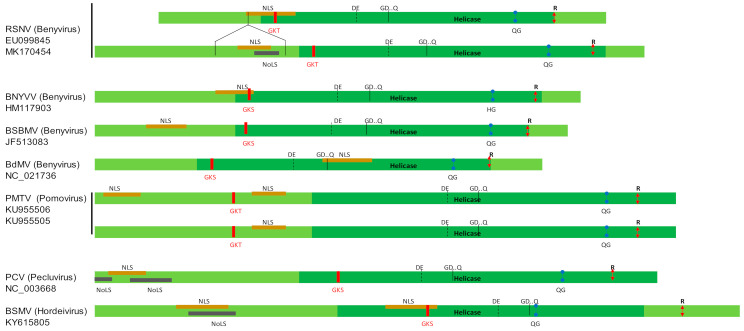
Organization of benyvirus and hordei-like TGBp1. Nucleolar localization signal (NoLS) and nuclear localization signals (NLS) were detected using Nucleolar localization sequence detector (NOD) [44] and cNLS mapper, for prediction of NLS specific to the importin αβ pathway [45]. Helicase domain and its conserved motifs I-VI were indicated in dark green based on [46]. The RSNV insertion domain is indicated by black lines. RSNV, rice stripe necrosis virus isolates col and M1-1, BNYVV, beet necrotic yellow vein virus, BSBMV, beet soil-borne mosaic virus, BdMV, burdock mottle virus, PMTV, potato mop-top virus, PCV, peanut clump virus, BSMV, barley stripe mosaic virus.

There were no insertion-deletion events in RNA1. Compared to the publicly available sequences, the RNA2 of all the new isolates showed a longer sequence due to an in-frame insertion of 243 nucleotides length at the position 252 of the tgbp1 gene (corresponding to position 2444 in the RNA2 segment). This insertion was also observed in the UFGEm/2020-1 sequence from Brazil. The 81 amino acids inserted into the N-terminal part of TGBp1 did not show any homology with sequences available in the GenBank database, or in known conserved domains (data not shown). The analysis of the hydrophobic amino acid clusters revealed that this domain was absent from the BNYVV TGBp1 (Figure S1). The insertion contains a low number of hydrophobic amino acid clusters, a high number of charged residues (19), and patches of glycine (7), serine (12) and proline (5). These features would result in a second disordered domain in the RSNV TGBp1, as revealed by the PONDR^®^ VL-XT analysis (Figure S2). Moreover, in addition to a NLS found in all RSNV TGBp1, a NoLS has been detected exclusively in the additional sequence (Figure 2).

The RSNV sequences were highly conserved (>97.5% sequence identity). However, the overall mean distances of the 16 RSNV genomes were higher than expected in comparison to the nine BNYVV genomes (0.023 subst/nt ± 0.002 and 0.025 subst/nt ± 0.002 for RSNV RNA1 and RNA2 compared to 0.007 subst/nt ± 0.001 and 0.014 subst/nt ± 0.004 for BNYVV RNA1 and RNA2, respectively). It is worth noting that the value from RSNV RNA1 was three times higher than from BNYVV. The genetic variability was differentially distributed along the two viral segments (Figure 1). In RSNV RNA1, genetic regions of Mtr, of Pro and between Pro and RdRp showed the highest variability at synonymous and non-synonymous levels. Significantly positive Tajima’s D values were observed in these domains (Figure S3). The genetic diversity of RSNV and BNYVV RNA2 showed less contrasted patterns. Synonymous and non-synonymous substitutions were homogeneously distributed in the cp-rt gene. The number of non-synonymous changes was low in the tgbp and crp genes.

**Figure 3:**
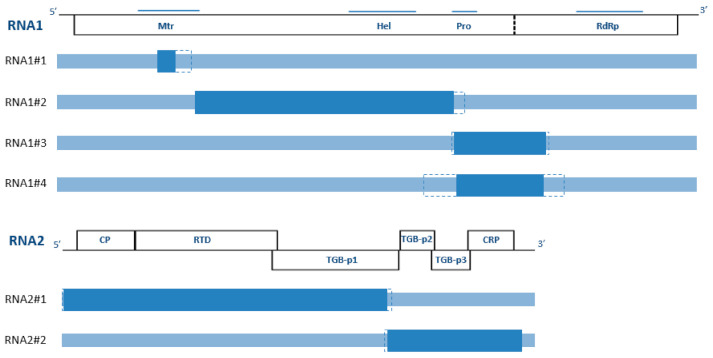
Recombination in RSNV genome. Genetic organization of the two RNA segments is indicated as in Figure 1. Dark blue rectangles indicate the location of the recombination events using RDP v4.974 [48] and dotted lines indicate the 99% of confident intervals. Five recombination events have been identified in this study.

Recombination detection was performed using the RDP4 software (Figure 3, Table 2). For the first time in the genus *Benyvirus*, statistically significant evidence for recombination was detected in both segments (3.42 × 10^−37^ < P < 4.67 × 10^−4^). Four and two putative recombination events were identified in RNA1 and RNA2, respectively. In RNA1, putative recombinant events were located in the Mtr region (isolate M1-1), between Mtr and Pro regions (isolate SL254) and between the Pro and RdRp regions (isolates col & Be2). In RNA2, the putative recombinant events concerned one or the other part of the two-thirds of the segment (isolates Be2, BR-LE01-19 and col). The same event RNA2#2 was detected in isolates BR-LE01-19 and col. One of the two putative parents was still unknown in 4/6 recombination events.

**Figure 4:**
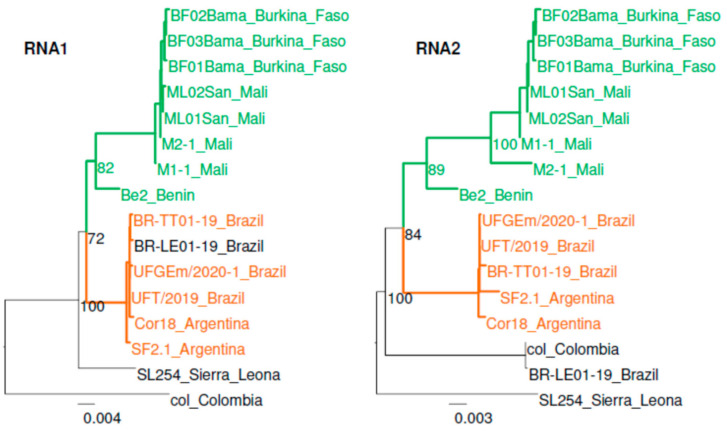
Phylogenetic relationships between RSNV genomic sequences. Maximum-likelihood (ML) phylogenetic trees were reconstructed with the sequences of the segments RNA1 and RNA2 of 16 RSNV isolates and without the recombinant regions. The trees were reconstructed using the PHYML-3.1 algorithm implemented in SEAVIEW v4.7 [39] under the best-fitted nucleotide substitution model (TN93+G) with gap treatment by pairwise deletion and with 100 bootstrap replications. The sequences clustering in the African and south African lineages are indicted in green and brown, respectively.

Phylogenetic analyses were performed without the recombinant domains (Figure 4). Two main lineages were detected, both with RNA1 and RNA2: one African lineage with isolates from Mali and Burkina Faso, and one South American lineage with isolates from Argentina and Brazil. This result contrasted with BNYVV for which no geographical structure has been detected between the previously reported types A and B [27] based on the phylogenetic trees with RNA1 and RNA2 (data not shown). The RSNV Colombian isolate, used as a reference until now, and the isolate SL254, showed a high divergence compared to the other isolates, suggesting the existence of other lineages. As described for BNYVV RNA3, 4 and 5 segments [28], we detected a putative reassortment event between RSNV RNA1 and RNA2. Indeed, RNA1 of the Brazilian isolate BR-LE01-19 belonged to the South American lineage, whereas its RNA2 was closer to the Colombian one. In addition, we noticed that although the RNA2 sequences of the Brazilian isolates BR-TT01-19 and UFT/2019 contained the same deletion in TGBp1, such as isolates BR-LE01-19 and col, they still clustered in the South American lineage.

Lineage-specific amino-acids were investigated using DNAsp software (Figure 1, Tables S2 and S3). Twenty-six specific residues were identified in each RNA. In RNA1, non-synonymous parsimony informative sites were located mainly in the Mtr or Pro regions, or between Pro and RdRp (9, 5 and 5 mutations, respectively). Most identified residues were specific to the African lineage, except for isolates M1-1 and Be2. In RNA2, non-synonymous parsimony informative sites were located mainly in CP-RTD and TGBp1 and, to a lesser extent, in TGBp3 and CRP (16, 5, 3 and 2, respectively). However, only three African lineage specific sites were identified at position 346 in CP-RTD, at position 107 in TGBp1 and at position 79 in CRP, with the exception of the isolate Be2.

## 4. Discussion

The genetic diversity data in the genus *Benyvirus* were mainly restricted to the smallest RNA segments of BNYVV, additionally to its cp gene. Until recently, only four whole genomes of RSNV from South America have been available in the public databases, regardless of its wide distribution in Africa. In this study, we used a combination of three sequencing techniques to obtain the first corpus of African genomic sequences of RSNV. Genomic data thus increased to 16 sequences, including two isolates from Argentina. Firstly, we revealed a new feature in RSNV RNA2. All the new sequences and the sequence UFGEm/2020-1 showed a conserved but longer TGBp1 compared both to the other benyvirus sequences, and to the other RSNV sequences from Colombia and Brazil. The 81 amino acids inserted into the N-terminal part of the protein would result in intrinsically disordered (ID) domains (Figure S2). ID domains are frequent in viral proteins and can be involved in RNA interaction [49,50]. Intrinsic disorder has been previously reported in the N-terminal part of the barley stripe mosaic virus TGBp1, a hordeivirus [49]. Interestingly, RSNV and Hordei-like TGBp1 share another common feature: the presence of putative NoLS and NLS, both located in the RSNV insertion (Figure 2). Similar to animal viruses, NoLS and nucleolus have been pointed out as playing a role in several plant-virus interactions [51]. Recently, Li et al. [52] have shown that the barley stripe mosaic virus TGBp1 hijacks the nucleolar protein Fibrillarin (Fib2) for cell-to-cell movement of the virus. Fib2 has also been shown to play a role in the long-distance movement of the rice stripe virus [53]. Further investigations should be performed to evaluate the functional role of this insertion in TGBp1. Notably, the absence of 81 residue-long insertions in the RSNV reference master genome from Colombia results in truncated sequences due to unmapped reads from the Illumina sequencing. The availability of new genomic data should now allow a reanalysis of the raw data, and to check the *tgbp1* gene length in the other sequences from Brazil. In addition, even if the isolate col has been sequenced using the Sanger method, we would suggest re-sequencing to confirm the absence of the insertion in the *tgbp1* gene. The collection and sequencing of other contemporaneous and/or sympatric isolates in Colombia would also be very interesting in order to decipher the evolutionary pathway of the TGBp1 indel. We noticed that the reference isolate col is one of the most divergent of the other isolates, even in RNA1, or outside TGBp1 in RNA2 (Figure 4).

The genetic diversity of the RNA1 and RNA2 showed contrasted characteristics depending on the benyvirus studied. Surprisingly, RNSV RNA1 showed a diversity index three times higher than expected, based on BNYVV data [29]. We detected significant positive Tajima’s D values in the Mtr region, and in the genetic domain between Pro and RdRp, suggesting a balancing selection in RSNV RNA1 only. Clusters of RSNV-specific polymorphisms were identified in these domains. In addition to a comparison of RSNV/BNYVV, further investigations of the diversity of BNYVV housekeeping genes should be performed, to better understand its evolution. In our study, the low nucleotidic diversity, the detection of putative recombination events, and the limited number of RSNV sequences, still make reassortment detection difficult. Nevertheless, the phylogeny obtained with the two RNA segments of the Brazilian isolate BR-LE01-19, suggests a reassortment event as previously reported for BNYVV RNA3, 4 and 5 segments [28] or as shown between BNYVV and Beet soil-borne mosaic virus RNA1 and RNA2 [21]. By using some of our sequences publicly available, de Souza et al. (2021) have also proposed such a reassortment [54]. Such a possibility remains to be carefully verified, since at this stage one cannot rule out the possibility of simultaneous co-occurrences. As genetic reassortment potentially results in fitness advantages or disadvantages to the progeny virus [55], the viral life traits of this Brazilian RSNV isolate should also be compared to non-reassortant ones.

In the genus *Benyvirus*, inter-species recombination has been frequently discussed in connection with the co-occurrence of BNYVV and BSBMV [28]. However, the detection of intra-species recombination events has been poorly investigated, probably due to the lack of whole genome sequences, the low intra-species genetic diversity and the focus on RNA3 diversity. In addition, frequency of recombination appears to be much lower in segmented compared with multipartite viruses [55]. In this study, by increasing the amount of genetic data, we revealed the first recombination events in the RSNV genome. As expected, the inferred phylogenetic relationships of putative recombinant isolates were different, with or without recombinant domains (Figure S4). It is worth noting that the RNA1 phylogeny of the putative recombinant isolate M1-1 was maintained, probably due to the smaller size of the recombinant domain. In addition, we performed complementary phylogenetic analyses with partial publicly-available sequences of other isolates. Phylogenetic trees inferred from the helicase region in RNA1 and the *cp* gene in RNA2, revealed new putative variants in Burkina Faso (isolates BF-K1 and BF-VK1) and Mali (isolates MALI-B1, MALI-I1 and MALI-SK1) (Figure S5). Validation should be performed by the entire sequencing of these isolates and by recombination analyses. In conclusion, this study allowed (i) the identification of RSNV domains showing molecular evolution signatures with putative functional impacts and (ii) the definition of two main RSNV lineages emerging in America and re-emerging in Africa, and several putative recombinant isolates. New sequence data on benyviruses will be required to identify parental lineages, and to reconstruct the evolutionary history of the two RNA segments, as an aspect which appears more complex than previously thought.

## Figures and Tables

**Table 1:** List of RSNV isolates used in this study with origin, collection date, accession numbers and bibliographic reference.

Country	Date	Name	Accession Numbers	Reference
Argentina	2017	Cor4	Hel MG792545	[11]
Argentina	2017	STFe5	Hel MG792546	[11]
Argentina	2017	STFe10.1	Hel MG792544	[11]
Argentina	2017	SF2.1	ARN1 MW147224, ARN2 MW147225	This study
Argentina	2018	Cor18	ARN1 MW147222, ARN2 MW147223	This study
Benin	2013	Be1	Hel KP099623	[4]
Benin	2013	Be2	ARN1 MW187590, ARN2 MW187591	This study
Brazil	2019	UFT/2019	ARN1 MT027255, ARN1 MT027256	Direct submission
Brazil	2019	BR-LE01-19	ARN1 MT270127, ARN2 MT270128	Direct submission
Brazil	2019	BR-TT01-19	ARN1 MT270129, ARN2 MT270130	Direct submission
Brazil	2020	UFGEm/2020-1	ARN1 MT507288, ARN2 MT507289	Direct submission
Burkina Faso	2013	BFRS1	CP LK023710	[3]
Burkina Faso	2016	BF-VK1	Hel MF115599, CP MF115604	[5]
Burkina Faso	2016	BF-K1	Hel MF115600, CP MF115605	[5]
Burkina Faso	2016	BF01Bama	ARN1 MW117944, ARN2 MW117939	This study
Burkina Faso	2016	BF02Bama	ARN1 MW117945, ARN2 MW117940	This study
Burkina Faso	2016	BF03Bama	ARN1 MW117946, ARN2 MW117941	This study
Colombia	2007	col	ARN1 EU099844, ARN2 EU099845	[9]
Mali	2015	MALI-SK1	Hel MF115602, CP MF115607	[5]
Mali	2015	MALI-I1	Hel MF115603, CP MF115608	[5]
Mali	2016	MALI-B1	Hel MF115601, CP MF115606	[5]
Mali	2016	M1-1	ARN1 MK170452, ARN2 MK170454	Direct submission
Mali	2016	M2-1	ARN1 MK170453, ARN2 MK170455	Direct submission
Mali	2016	ML01San	ARN1 MW117948, ARN2 MW117943	This study
Mali	2016	ML02San	ARN1 MW117947, ARN2 MW117942	This study
Sierra Leona	2019	SL254	Hel MN750254, CP MN750255, ARN1 MW187592, ARN2 MW187593	[6] This study

**Table 2:** Recombination events detected in RSNV complete genome.

Recomb. Event ^a^	Recombinant ^a^	Major Parent	Minor Parent	Beginning Breakpoint ^b^	Ending Breakpoint	Positive Methods ^c^	*P* Value
RNA1 #1	M1-1	BF01Bama	Cor18	1026 (nt)	1213 (1129–1375)	G,B,M,C,T	1.24 × 10^−5^
RNA1 #2	SL254	SF2.1	unknown	1392 (nt)	4097 (2818–4208)	R,M,C,S,T	4.49 × 10^−5^
RNA1 #3	col	unknown	BF02Bama	4109 (4081-4148)	5063 (4999–5093)	R,G,B,M,C,S,T	3.42 × 10^−37^
RNA1 #4	Be2	M1-1	Cor18	4121 (3787–4208)	5031 (4826–5228)	G,B,M,C,S	4.67 × 10^−4^
RNA2 #1	Be2	unknown	BF02Bama	16 (1–354)	3360 (3337–3410)	R,M,S,T	4.18 × 10^−13^
RNA2 #2 *	BR-LE01-19	unknown	BF02Bama	3360 (3322–3586)	4751 (nt)	R,G,B,M,C,S,T	3.88 × 10^−12^

^a^ as labeled in Figure 3, ^b^ the 99% confident intervals between brackets, nt: not determined, ^c^ algorithms implemented in RDP v4.974 software [48]: R, RDP, G, GeneConv, B, Bootscan, M, MaxChi, C, Chimaera, S, Siscan, T, 3Seq, * the same putative recombinant event was found in two sequences: BR-LE01-19 and col.

## Data Availability

Nucleotide sequence data reported are available in the GenBank database under the accession number(s) MW117939-MW117948, MW147222-MW147225, MW187590-MW187593.

## Supplementary Materials

The following are available online at https://www.mdpi.com/article/10.3390/v13050737/s1, Figure S1: Hydrophobic cluster analyses on TGBp1 proteins of BNYVV (isolate S) and RSNV (isolates col and M1-1), Figure S2: Predicted disordered segments in TGBp1 protein of RSNV isolate M1-1 using Predictor of Natural Disordered Regions with VL-XT algorithm (available at pondr.com), Figure S3: Tajima’s D (DT) values along the genomic sequences of RSNV and BNYVV using DNAsp software with default parameters, Figure S4: Maximum-likelihood phylogenetic trees reconstructed with the 16 RSNV complete genome sequences (RNA1 and RNA2) with or without the recombinant regions, Figure S5: Maximum-likelihood phylogenetic trees reconstructed with the 16 RSNV complete genome sequences (without the recombinant regions) and with the helicase region or the cp gene, Table S1: Primers used for amplification of RSNV isolates, Table S2: Polymorphism analyses of BNYVV and RSNV sequences (RNA1 and RNA2), Table S3: Amino acid polymorphisms in RSNV and BNYVV proteins identified as parsimony informative.
