# Supplementary material for: Genetic Diversity of Rice stripe necrosis virus and New Insights into Evolution of the Genus Benyvirus
Source: Viruses. 2021 Apr 23;13(5):737. doi: 10.3390/v13050737 (PMC8145960; doi:10.3390/v13050737)
Supplement: Supplementary file 1 [file viruses-13-00737-s001.zip › viruses-1170742-SI.pdf]

Table S1. Primers used for amplification of RSNV isolates.

Isolates	Primers	Sequences	Segments
Be1	RSNV3'_ARN1	ATACAACTCACACTCAGTACACC	RNA1
	RSNV5'_ARN1	AATCGGACCGACCCCGGGAG	
	RSNV3'	ATACTGATACAACTCACAC	RNA2
	RSNV5'_ARN2	GACGAATTCCACCTACG	
SF2.1<br>SL254	RSNV5'_ARN1	AATCGGACCGACCCCGGGAG	RNA1
	RSNV1-0511R	AAGCCTGCAGTGTCGACATA	
	RSNV1-1097F	AATATGAACGTGTGGCTTCAG	
	RSNV1-2021R	TCTTTCGGCAATAGCAACAC	
	RSNV1-2901F	TGAATTTGGTGCTCTCTTG	
	RSNV1-3126-R	AGCTTTGTGTTGGGTATGA	
	RSNV1-3573-F	GACGGTGCGATCTAATCAAGGTTCTAC	
	RSNV1-3827R	TGTGGCGTTTCCAGACCTAAA	
	RSNV1-4337R	TGAGGCTTGGGCTTAACATAAC	
	RSNV1-5080F	CGTACAGATCTCTTATGGTTGGT	
	RSNV1-5186R	GTAACAAAGTTAGTGGCAACC	
	RSNV1-6116F	CCCTCAGAGATTGGTTGAACAG	
	RSNV3'_ARN1	ATACAACTCACACTCAGTACACC	
	RSNV2-5prima	TATCACTACTGACGAATTCCACCTAC	RNA2
	RSNV2-1116F	CCACCACGTTATCATGTTCCT	
	RSNV2-1223R	AATCTGCGGCCTGTTTTGTA	
	RSNV2-2286F	CTCCCCATGTCGTCAACTTT	
	RSNV2-2344R	CCTCGTTTTCAAGAAGATCG	
	RSNV2-3352F	AACTCATTCCGCTGGTGAT	
	RSNV2-3461R	GACATTTGCGGTTGAGAGGA	
	RSNV2-3prima	TTTTTTTTTTTTTCCCGGATAGTAAACCTC	
BF01Bama<br>BF02Bama<br>BF03Bama<br>ML01San<br>ML02San<br>M1-1<br>M2-1	RSbegF	TGGATCCCATAGACGAAACGAAA	RNA1
	RS1607R	AATTTGAGGGGACATCCG	
	RS1293F	CCCCGACCATTTTCTATTT	
	RS2840R	CGTCTATCTCCTTCTGGG	
	RS2382F	CGATGCCACCAAAAAGAAC	
	RS4156R	ATGCAGTCGTCCCATGTTGA	
	RS3807F	TTTAGGTCTGGAAACGCCACA	
	RS5347R	GCCAGAGTTTATCGCTTTCA	
	RS5020F	AATTTGAGGGGACATCCG	
	BenydT-3’	GGATCCCGGGTTTTTTTTTTTTTTTTGTRTAT	
	RS2begF	GGCATATCACTACTGACGAATTCCAC	
	RS2-1697R	GCCGCAGTCCTATCTCCTTG	
	RS2-1485F	AAGATGTGGCGGGACGTT	RNA2
	RS2-2475R	AGAGTAGTCTTTCCCGCACCG	
	RS2-2315F	TGGAGAAGAGCAAAGCTATAAG	
	RS2-3204R	GTTTGCCCACTACATGAAGAA	
	RS3031F	TTTGCTGCAGTTACTCTCATT	
	BenydT-3’	GGATCCCGGGTTTTTTTTTTTTTTTTGTRTAT	

Table S2. Polymorphism analyses of BNYVV and RSNV sequences (RNA1 and RNA2)

BNYVV	ORF1	CP-RTD	TGBp1	TGBp2	TGBp3	CRP
Number of sites	6330	2076	1155	357	399	390
Variable (polymorphic) sites	166	154	51	21	23	24
Singleton variable sites	144	63	21	12	11	14
Parsimony informative sites	22	91	30	9	12	10
Total nb of syn. changes	123	118	44	14	14	14
Total nb of replacement changes	45	39	7	7	9	10
Nb of syn. informative sites	18	74	29	7	9	6
Nb of non-syn. informative sites	4	17	1	2	3	4

RSNV	ORF1	CP-RTD	TGBp1	indel*	TGBp2	TGBp3	CRP
Number of sites	6303	2019	1293	243	354	402	462
Variable (polymorphic) sites	501	176	92	24	21	20	27
Singleton variable sites	293	70	33	10	7	7	12
Parsimony informative sites	208	106	59	14	13	13	14
Total nb of syn. changes	429	126	85	16	19	15	23
Total nb of replacement changes	65	56	8	6	2	5	4
Nb of syn. informative sites	181	90	58	11	13	9	12
Nb of non-syn. informative sites	27	16	1	3	0	4	2

*analysis performed with the RSNV sequences showing the insertion on TGBp1

Table S3. Amino acid polymorphisms in RSNV and BNYVV proteins identified as parsimony informative.

RNSV RNA1					Mtr										Hel	Pro										RdRp	
Residue	aa	11	68	223	309	322	328	329	333	338	343	345	362	826	1089	1332	1334	1337	1362	1364	1435	1463	1466	1479	1568	1821	1844
		L/P	K/E	L/F	R/K	T/S	R/P	V/L	D/H	V/M	G/S	A/G	A/P	I/T	I/V	I/V	A/D	T/A	R/Q	Q/N	A/V	A/V	R/G	E/G	V/I	S/F	T/I
Burkina Faso	BF01Bama	L	K	F	K	S	P	L	H	M	S	G	P	I	I	I	A	T	R	Q	A	A	R	E	V	F	I
	BF02Bama	L	K	F	K	S	P	L	H	M	S	G	P	T	I	I	A	T	R	Q	A	A	R	E	V	F	I
	BF03Bama	L	K	F	K	S	P	L	H	M	S	G	P	I	I	I	A	T	R	Q	A	A	R	E	V	F	I
Mali	ML02San	L	E	F	K	S	P	L	H	M	S	G	P	T	I	I	A	T	R	Q	A	A	R	E	V	F	I
	ML01San	L	E	F	K	S	P	L	H	M	S	G	P	T	I	I	A	T	R	Q	A	A	R	E	V	F	I
	M2-1	L	E	F	K	S	P	L	H	M	G	G	P	I	I	I	A	T	R	Q	A	A	R	E	V	F	I
	M1-1	L	K	L	R	T	R	V	D	V	G	A	A	I	I	I	A	T	R	Q	A	A	R	E	V	S	T
Benin	Be2	L	K	L	R	T	R	V	D	V	G	A	A	I	V	I	D	A	Q	N	V	V	G	G	I	S	T
Sierra Leone	SL254	L	K	L	R	T	R	V	D	V	S	A	A	I	V	V	D	A	Q	N	V	V	G	G	I	S	T
Argentina	Cor18	L	K	L	R	T	R	V	D	V	G	A	A	I	V	V	D	A	Q	N	V	V	G	G	I	S	T
	SF2.1	L	K	L	R	T	R	V	D	V	G	A	A	I	V	V	D	A	Q	N	V	V	G	G	I	S	T
Brazil	UFT/2019	L	K	L	R	T	R	V	D	V	G	A	A	I	V	V	D	A	Q	N	V	V	G	G	I	S	T
	BR-TT01-19	P	K	L	R	T	R	V	D	V	G	A	A	I	V	V	D	A	Q	N	V	V	G	G	I	S	T
	UFGEm-2020	L	K	L	R	T	R	V	D	V	G	A	A	I	V	V	D	A	Q	N	V	V	G	G	I	S	T
	BR-LE01-19	P	K	L	R	T	R	V	D	V	G	A	A	I	V	V	D	A	Q	N	V	V	G	G	I	S	T
Colombia	col	L	K	L	R	T	R	V	D	V	G	A	A	I	V	I	A	T	Q	Q	A	A	R	E	V	S	T

RSNVRNA2		CP-RTD																		TGBp1			TGBp3			CRP	
Residue	aa	30	59	152	180	188	222	255	346	417	435	465	482	514	573	606	631	107	108	133	162	413	15	16	17	79	85
		S/T	E/A	S/P	A/V	A/V	Q/R	P/L	K/E	F/L	S/G	N/S	K/R	A/S	P/T	P/S	S/G	I/V	S/P	A/T	S/G	D/N	C/Y	D/V	H/T	N/H	N/S
Burkina Faso	BF01Bama	T	A	S	V	V	Q	P	K	F	S	N	K	S	T	P	S	I	S	A	S	N	C	D	H	N	N
	BF02Bama	S	A	S	V	V	Q	P	K	F	S	N	K	S	T	P	S	I	S	A	S	N	C	D	H	N	N
	BF03Bama	T	A	S	A	V	Q	P	K	F	S	N	K	S	T	P	S	I	S	A	S	N	C	D	H	N	N
Mali	ML01San	T	A	S	A	V	Q	P	K	F	S	N	K	S	T	P	S	I	S	A	S	N	C	D	H	N	N
	ML02San	T	A	S	A	V	Q	P	K	F	S	N	K	S	T	P	S	I	S	A	S	N	C	D	H	N	N
	M1-1	T	E	P	V	A	Q	P	K	F	S	N	K	S	T	P	S	I	S	A	S	N	C	D	H	N	N
	M2-2	S	E	P	V	A	Q	P	K	F	S	N	K	A	P	S	G	I	P	T	S	D	C	D	H	N	N
Benin	Be2	T	E	S	A	A	Q	P	E	F	S	N	K	A	P	P	G	V	S	T	S	D	Y	V	T	H	S
Sierra Leone	SL254	T	E	S	V	A	Q	P	E	F	S	N	K	A	P	S	S	V	S	T	S	D	Y	V	T	H	S
Argentina	SF2.1	T	E	S	A	A	R	P	E	F	S	N	K	A	P	S	G	V	P	T	G	D	Y	V	T	H	S
	Cor18	T	E	S	A	A	R	P	E	F	S	N	K	A	P	S	G	V	P	T	G	D	Y	V	T	H	S
Brazil	UFT/2019	T	E	S	A	A	R	P	E	F	S	N	K	A	P	S	G	-	-	-	-	D	Y	V	T	H	S
	BR-TT01-19	T	E	S	A	A	R	P	E	F	S	N	K	A	P	S	G	-	-	-	-	D	Y	V	T	H	S
	UFGEm-2020	T	E	S	A	A	R	P	E	F	S	N	K	A	P	S	G	V	P	T	G	D	Y	V	T	H	S
	BR-LE01-19	T	E	S	A	A	Q	L	E	L	S	S	R	A	S	S	G	-	-	-	-	D	C	D	H	H	N
Colombia	col	T	E	S	A	A	Q	L	E	L	G	S	R	A	S	S	G	-	-	-	-	D	C	D	H	H	N

BNYVV			Hel		CP-RTD																		TGB p1	TGBp2			TGBp3		CRP			
residue	121	467	1068	1511	62	102	172	238	291	296	327	332	348	350	361	369	447	500	502	583	593	622	110	97	100	44	91	130	1	45	68	75
	S/T	I/V	R/K	K/R	T/S	S/N	L/F	V/I	V/I	A/T	D/G	M/T	A/V	S/P	T/A	A/V	V/I	A/V	A/T	G/S	T/A	-/N	M/T	V/L	S/L	V/I	D/E	C/Y	M/T	Y/F	M/V	R/Q
US	T	I	R	K	T	S	L	V	V	A	D	M	A	S	T	A	V	A	A	G	T	-	M	V	S	V	D	C	M	Y	M	R
Braz	T	I	R	K	T	S	L	V	V	A	D	M	A	S	T	A	V	A	A	G	T	-	M	V	S	V	D	C	M	Y	M	R
Sp-S8	T	I	K	K	T	S	L	V	V	A	D	M	A	S	T	A	V	A	A	G	T	-	M	V	S	V	D	C	M	Y	M	R
Sw-E12	T	V	K	K	T	S	L	V	V	A	D	M	A	S	T	A	V	A	A	G	T	-	M	V	S	V	D	C	M	Y	M	R
Yougos	T	V	R	K	T	S	L	V	V	A	D	M	V	S	T	A	V	A	A	G	T	-	M	V	L	V	D	C	M	Y	M	R
Fr-P	S	I	R	R	T	S	L	I	V	A	D	M	A	S	A	A	I	A	A	S	A	-	M	L	S	V	E	C	M	Y	M	R
Jap-S	S	I	R	K	T	S	L	I	V	A	D	M	A	S	A	A	I	A	A	G	A	-	M	L	S	V	E	C	M	Y	M	Q
Ch-Hu3	S	V	R	R	S	N	F	I	I	T	G	T	V	P	T	V	I	V	T	G	A	N	T	L	L	I	E	Y	T	F	V	Q
Fr-F13	S	I	R	K	S	N	F	I	I	T	G	T	V	P	A	V	V	V	T	S	A	N	T	L	S	I	E	Y	T	F	V	Q

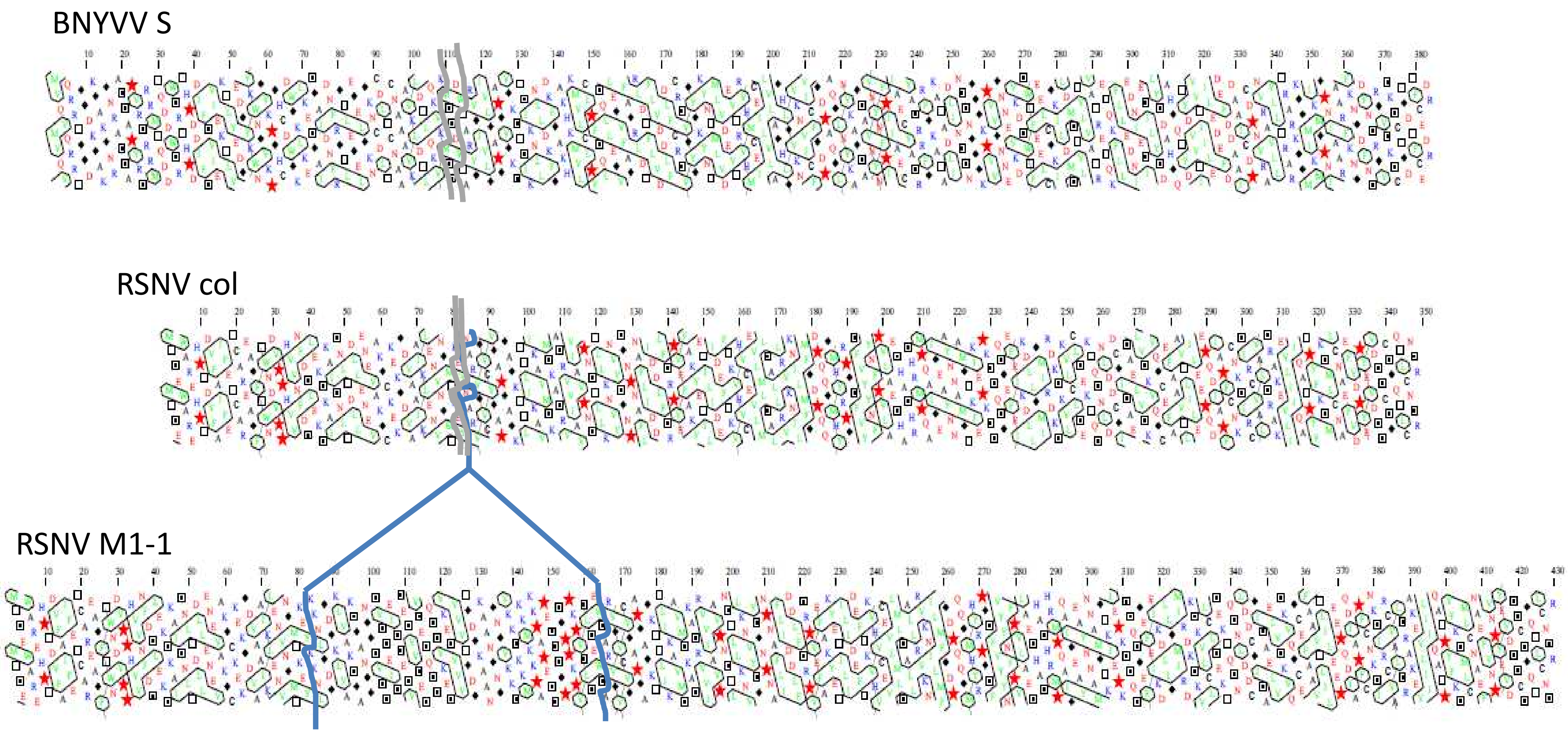

Figure S1. Hydrophobic cluster analyses on TGBp1 proteins of BNYVV (isolate S) and RSNV (isolates col and M1-1). As described in the original publication, this method relies upon a two-dimensional representation of the sequences [41]. The hydrophobic residues are encircled, and different symbols are used for prolines (red star) which are considered as breaker of hydrophobic clusters and glycines (diamond) which are often present in loops. The HCA plots were obtained at https://mobyle.rpbs.univ-paris-diderot.fr/cgi-bin/portal.py#forms::HCA. Plots are aligned and the insertion domain in the RSNV sequences is indicated by blue lines.

RSNV col

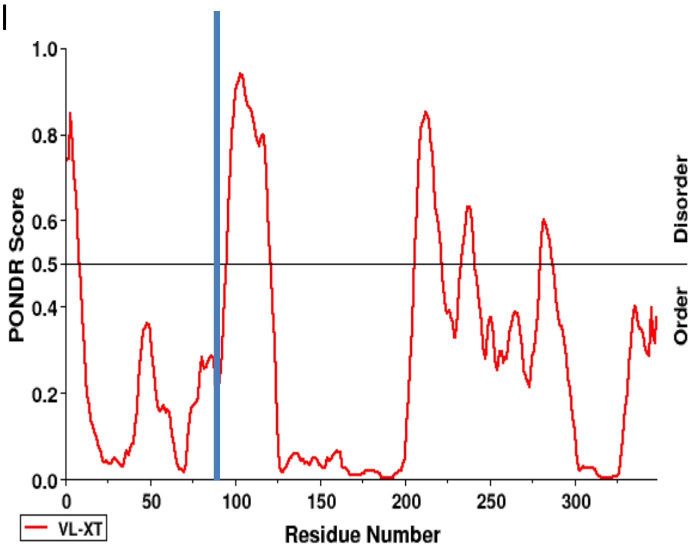


```
Disordered segment   95 AGKTTLIKAVMSKASRVVIAVPNSTL 120
                        DDDDDDDDDDDDDDDDDDDDDDDDDD
```

Average Strength 0.8019

RSNV M1-1

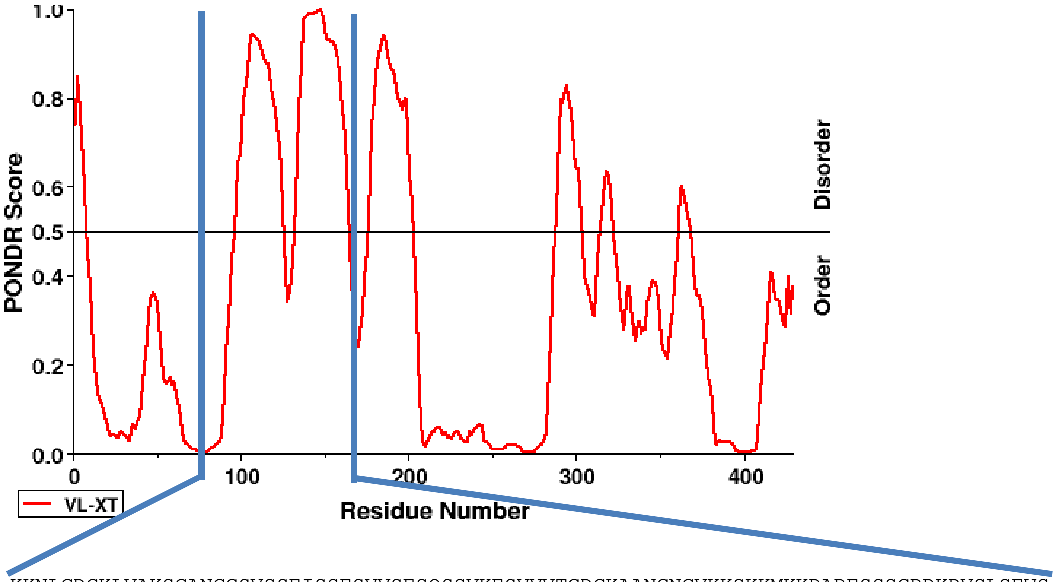


```
84 KKNLGDGKLVAKSGANGGSVSSEISSESVVSESQSSVKESVVVTGDGKAANGNGVKKSKKMKKPAPESSSGPPKPVSLSEWS 165
                DDDDDDDDDDDDDDDDDDDDDDDDDDDDD       DDDDDDDDDDDDDDDDDDDDDDDDDDDDDDDD
```

Disordered segment	[97]-[125]	[133]-[164]
Average Strength	0.8061	0.8863

Figure S2. Predicted disordered segments in TGBp1 protein of RSNV isolates col and M1-1 using Predictor of Natural Disordered Regions with VL-XT algorithm (available at pondr.com) [42,43]. Blue lines indicated the insertion site. The borders of the disordered segments and the average strength values were indicated below the amino acid sequence of the insertion.

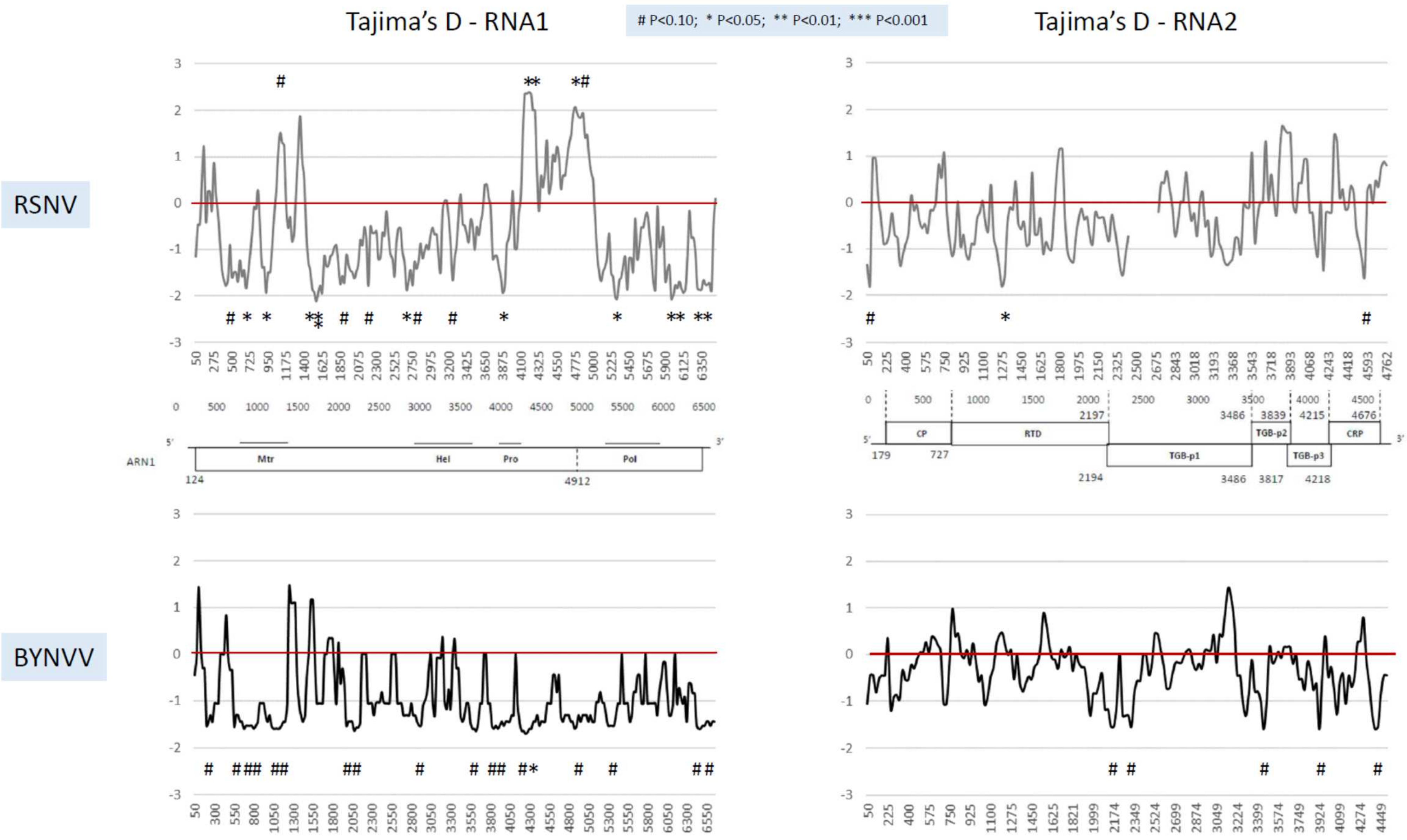

Figure S3. Tajima's D (DT) values along the genomic sequences of RSNV and BNYVV using DNAsp software with default parameters [47]. DT = 0 correspond to a mutation-drift equilibrium, DT > 0 indicates balancing selection, sudden population contraction and DT < 0 distinguish a recent selective sweep, population expansion after a recent bottleneck, (# P<0.10;* P < 0.05; ** P<0.01; ***P<0.001).

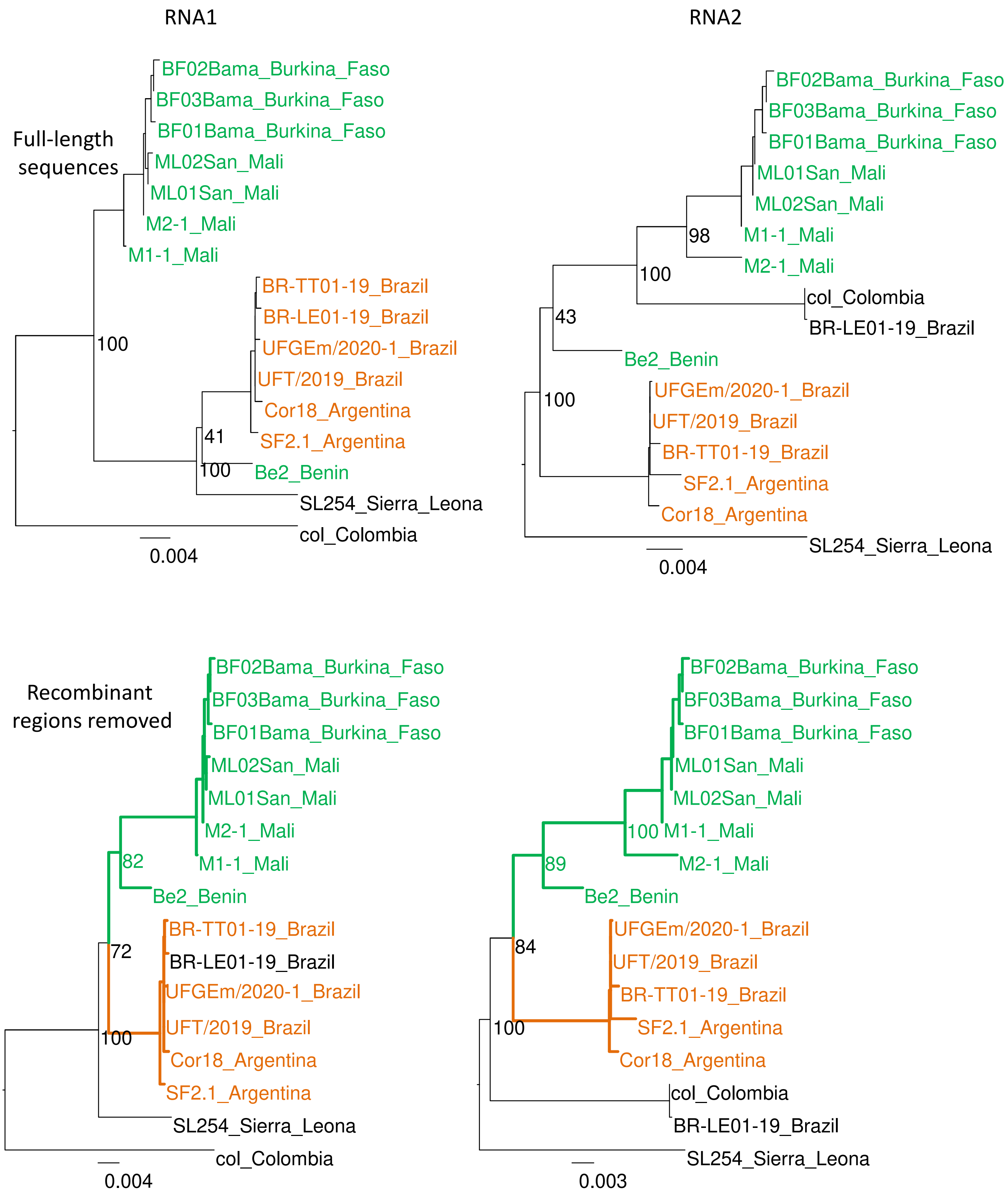

Figure S4. Maximum-likelihood phylogenetic trees reconstructed with the 16 RSNV complete genome sequences (RNA1 and RNA2) with or without the recombinant regions.

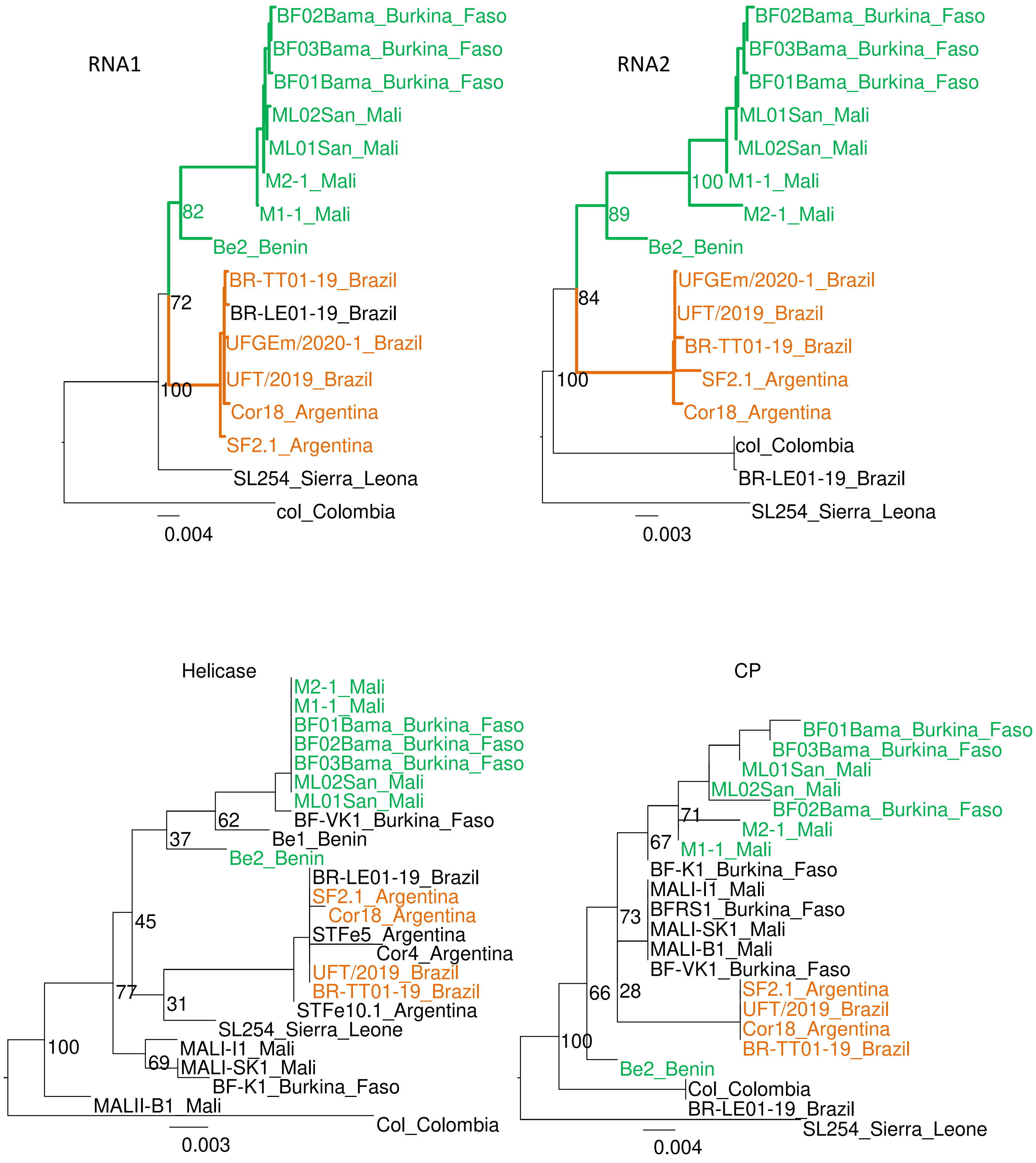

Figure S5. Maximum-likelihood phylogenetic trees reconstructed with the 16 RSNV complete genome sequences (without the recombinant regions) and with the helicase/CP genes.
